# Artificial intelligence integration in the drug lifecycle and in regulatory science: policy implications, challenges and opportunities

**DOI:** 10.3389/fphar.2024.1437167

**Published:** 2024-08-02

**Authors:** Wahiba Oualikene-Gonin, Marie-Christine Jaulent, Jean-Pierre Thierry, Sofia Oliveira-Martins, Laetitia Belgodère, Patrick Maison, Joël Ankri, The Scientific Advisory Board of ANSM

**Affiliations:** ^1^ Agence Nationale de Sécurité des Médicaments et des Produits de Santé (ANSM) Saint-Denis, Saint-Denis, France; ^2^ INSERM, Laboratoire d'Informatique Médicale et d'Ingénierie des Connaissances en e-Santé, LIMICS, Sorbonne Université, Paris, France; ^3^ France Assoc Santé, Paris, France; ^4^ Faculty of Pharmacy of Lisbon University, Lisbon, Portugal; ^5^ CHRC – Comprehensive Health Research Center, Evora, Portugal; ^6^ EA 7379, Faculté de Santé, Université Paris-Est Créteil, Créteil, France; ^7^ CHI Créteil, Créteil, France; ^8^ Université de Versailles St Quentin-Paris Saclay, Inserm U1018, Guyancourt, France

**Keywords:** artificial intelligence, health policy, regulatory science, drug lifecycle, drug approval process, patient safety

## Abstract

Artificial intelligence tools promise transformative impacts in drug development. Regulatory agencies face challenges in integrating AI while ensuring reliability and safety in clinical trial approvals, drug marketing authorizations, and post-market surveillance. Incorporating these technologies into the existing regulatory framework and agency practices poses notable challenges, particularly in evaluating the data and models employed for these purposes. Rapid adaptation of regulations and internal processes is essential for agencies to keep pace with innovation, though achieving this requires collective stakeholder collaboration. This article thus delves into the need for adaptations of regulations throughout the drug development lifecycle, as well as the utilization of AI within internal processes of medicine agencies.

## Introduction

The healthcare landscape has recently witnessed a proliferation of AI applications, many of which have found practical implementation through medical devices. These applications span various medical specialties, including radiology (Samala et al., 2016), dermatology (Esteva et al., 2017), ophthalmology (Abràmoff et al., 2018), pathology (Litjens et al., 2016), genome interpretation (Kamps et al., 2017), biomarker discovery (Diaz-Uriarte et al., 2022), and drug shortage studies (Pall et al., 2023). It is worthy to note, however, that for applications like radiology, for instance, the use of AI is still far from routine, and needs dedicated teams and skills (Shelmerdine et al., 2024). Furthermore, AI is making new inroads into clinical trial processes (EMA, 2022), with the recent milestone of the first wholly AI-designed drug (Chace, 2024). Although still in its nascent stages, the theoretical potential of AI in pharmaceutical product development is vast, spanning from rational drug design and decision-making support to personalized medication and clinical data management (Duch et al., 2007; Blasiak et al., 2020; D Amico et al., 2023). Consequently, AI tools and applications are poised to play an increasingly pivotal role across all stages of the drug lifecycle, including drug discovery, manufacturing, nonclinical testing, clinical research, and surveillance (Harrer et al., 2019; Gupta et al., 2021; Hauben and Hartford, 2021; Kang et al., 2023). This review elucidates the profound regulatory implications of AI’s existing or potential involvement in pharmaceutical product development at every stage of the drug lifecycle, particularly in relation to the body of evidence utilized for clinical trials and marketing authorization. As regulatory agencies are tasked with ensuring the quality, safety, and efficacy of medicinal drugs and are at the forefront of assessing these evolving methodologies, the overarching aim of this paper is to comprehensively explore the potential spectrum of AI applications in drug-related regulatory science with proposals for actionable regulatory recommendations. Additionally, this paper reviews the potential of AI to enhance and optimize regulatory processes at regulatory agencies concerning drug assessment, authorization, and post-authorization surveillance.

We will first give an overview of existing or potential AI applications in the drug lifecycle, with step-specific questions about the data and models used and the corresponding regulatory challenges and policy implications. In a second part, we will propose regulatory recommendations or adaptations that may be required to meet those challenges. In a third part, we will show how AI may help optimize and expedite internal regulatory agencies’ processes, to the benefit of patients. We hope that this perspective will contribute to accelerating relevant future regulatory adaptations and understanding among all stakeholders in the field of AI use in the drug lifecycle.

### Policy implications regarding stepwise AI applications in the drug lifecycle

The potential uses of AI are outlined here across different phases of the drug life cycle, from drug discovery to clinical trials and post-authorization activities.

AI algorithms are widely applied for drug discovery (Burki, 2019; Vamathevan et al., 2019). Quantitative structure-activity/property relationship (QSAR/QSPR), as well as structure-based modeling, new molecule design, and synthesis prediction, may be addressed by AI (Jiménez-Luna et al., 2021; Paul et al., 2021; Vora et al., 2023). Computational methods have been used for a long time for ligand-binding probability calculations (Fujita and Winkler, 2016) and for ADMET (absorption, distribution, metabolism, and toxicity) prediction (Norinder and Bergström, 2006; Beck and Geppert, 2014). Several pharmaceutical companies are currently working with AI organizations (such as companies and research laboratories) along different lines (Paul et al., 2021). Recently, the first wholly AI-designed drug entered clinical trials (Chace, 2024). During the development of this new drug, TRAF2- and NCK-interacting kinase (TNIK) was first identified as an anti-fibrotic target using a predictive artificial intelligence (AI) approach (using PandaOmics (Kamya et al., 2024)). Then, using generative AI [Chemistry42 (Ivanenkov et al., 2023)], a small-molecule TNIK inhibitor was designed (Ren et al., 2024). This drug entered two phase I studies in 18 months, from target discovery to preclinical candidate, including traditional testing in animal models, which is a very short timeline. Regarding timelines and costs, it is usually around 5.5–14.5 years (or more for target discovery) without the AI approach to reach the preclinical stage. In terms of costs, the traditional approach costs around 674 million dollars for a preclinical candidate, whereas it is much lower with the AI approach (Pun et al., 2024). The application of AI in drug screening could reduce R&D costs by 50% while increasing efficiency and accuracy (Wang et al., 2019). As another example of a state-of-the-art recent AI application for drug discovery, AlphaFold allows predicting protein structures at the atomic level, potentially accelerating drug discovery in cancer research (Abramson et al., 2024; Xu et al., 2024). Nevertheless, even if all these technologies and their potentials seem impressive, most are at preliminary stages, there are few success stories, and it still remains to be determined if AI will really perform better and faster to develop more and more new successful drug candidates (Schneider et al., 2020; Bender and Cortés-Ciriano, 2021). Moreover, till now, in the cases reviewed above, preclinical validation was carried out in traditional animal models.

In addition to potentially helping predict toxicity of drug candidates, AI approaches in preclinical testing can contribute to replacing, reducing, and refining the use of animals (Luechtefeld et al., 2018). This second incentive is quite powerful. As in drug discovery, large amounts of toxicological data already exist and can be used to construct AI tools that are relevant for toxicity prediction (Mayr et al., 2016; Luechtefeld et al., 2018; Lysenko et al., 2018; Basile et al., 2019; Wu et al., 2021). Non-animal approaches (such as QSAR, read-across, PB/PK, metabolomics, and cell painting, to cite just a few) rely as well on big toxicological, biological, and chemical data (Bray et al., 2016; Luechtefeld et al., 2018; Liu et al., 2023), for which quality should be thoroughly checked and ensured before training any prediction model, given that new kinds of toxicity cannot always be derived from previously learned ones (reliance solely on historical toxicology data might not be sufficient in several cases).

In the future, AI tools might be used for improving clinical trials with digital twins and optimizing the control arms (EMA, 2022; Fountzilas et al., 2022; Askin et al., 2023). They might help in patient selection and monitoring (eligibility, suitability, motivation, empowerment, adherence, and retention), thereby increasing clinical trials’ success rates (Harrer et al., 2019). They could also participate in designing more relevant trials, especially for precision medicine (for a review, see (Fountzilas et al., 2022)). Patient selection is the area where AI could be most used, followed by trial design (two times less) and analysis (three times less) (Askin et al., 2023). Overall, it is the mass and diversity of data that AI can process that could make the difference. Biomedical data from different origins (such as health insurance medical records, hospitals, genomics, biobanks, and radiology) may indeed be used to improve the enrolment and the design and follow-up of clinical trials (Acosta et al., 2022). It is also used to generate synthetic clinical data (synthetic patients) for accelerating precision medicine, increasing the coverage of the population involved in the clinical trial (Yu et al., 2018; EMA, 2022).

AI may be used to improve quality-by-design approaches (Rantanen and Khinast, 2015; Manzano et al., 2021). This includes tools to deal with the interpretation of experimental big data from various sources, such as real-time process control and real-time quality assurance (Hussain et al., 1991; Takayama et al., 1999; Rantanen and Khinast, 2015).

Pharmacovigilance (PV) is a data-driven field because it necessitates the gathering, processing, and analysis of significant amounts of data from a variety of very different sources (Carbonell et al., 2015). Here, AI techniques may be used for signal detection, data intake, or analysis (Hauben and Hartford, 2021). In practice, it is used and recommended mostly for signal detection and processing before data intake (Ball and Dal Pan, 2022; Martin et al., 2022). Industrials have reported the performance of several AI systems for signal detection and adverse event processing (Schmider et al., 2019; Routray et al., 2020). One study showed that the use of safety database data fields with dedicated AI applications (artificial intelligence and robotic process automation) as a surrogate for otherwise time-consuming and costly direct annotation of source documents is viable and feasible (Schmider et al., 2019). An example of an augmented AI system with a neural network approach used for an accurate and scalable solution for pharmacovigilance determination of adverse event seriousness in spontaneous, solicited, and medical literature reports was published (Routray et al., 2020). Data from a wide variety of sources can theoretically be used, including real world data such as electronic healthcare records (EHR) or social media (Comfort et al., 2018; Ball and Dal Pan, 2022; Actualité, 2024). AI can also be used for finer drug misuse detection (Afshar et al., 2022).

## Actionable recommendations

### Regulatory agencies and stakeholders’ information needs- transparency & explainability

In the fast-paced world of drug development, transparency is a cornerstone of trust and accountability (Transparency, 2024). When it comes to the application of artificial intelligence (AI), transparency becomes even more crucial (Crossnohere et al., 2022). In this respect, it is of utmost importance that stakeholders – especially regulators –, have access to clear information about the AI models driving drug development. Goals, data used, intended applications, advantages, and drawbacks of AI models should be clear so that everyone understands how they fit into each specific drug development. Regulators need this level of transparency and explainability to assess accuracy, precision, limitations, and uncertainties effectively (Hicks et al., 2022). One of the answers is therefore explainable AI (Alizadehsani et al., 2024). This is a recent discipline by itself (xAI). Several mathematical techniques are used to render AI methods and results more easy to understand (reviewed in (Holzinger et al., 2022)). In this respect, techniques like SHapley Additive exPlanations (SHAP) and, Local Interpretable Model-agnostic Explanations (LIME), Integrated Gradients, and Counterfactual Explanations offer windows into the black box of AI decision-making, providing clarity on the processes behind the algorithms (Mertes et al., 2022; Kırboğa et al., 2023; Wang et al., 2024). And transparency doesn't end once the model is built. Since AI models may evolve, regulators need to stay in the loop on updates and changes, ensuring ongoing monitoring of their performance and impact.

**FIGURE 1:**
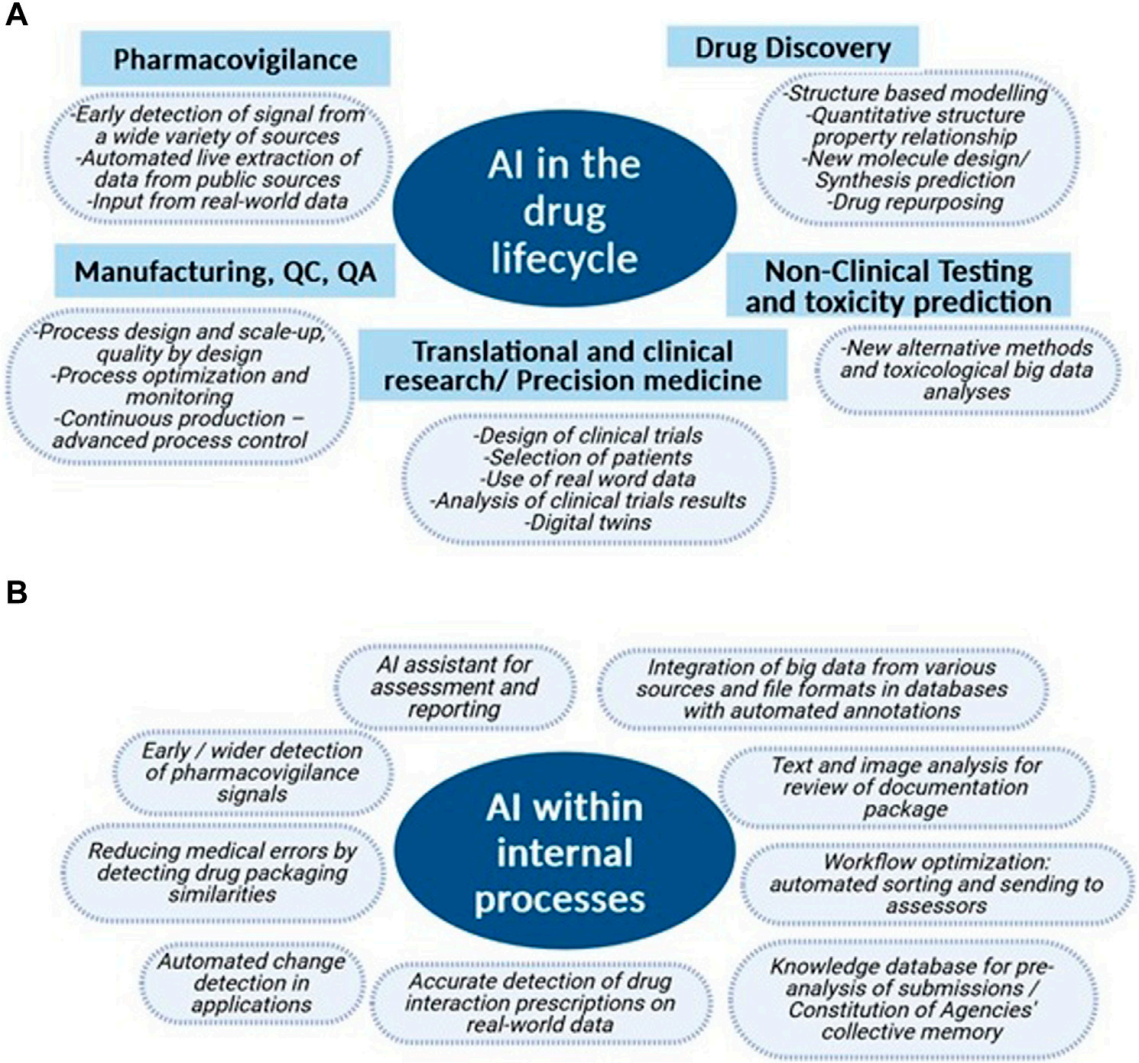
Domains of potential or existing use of AI in the drug lifecycle and for internal regulatory agencies’ processes. **(A)** Already existing or potential AI uses at each step of the drug lifecycle from drug discovery to post-market surveillance which are currently developed by researchers from academia and industry. **(B)** Examples of AI applications existing or in development in medicine agencies (in clockwise order of complexity, starting from *Integration of big data from various sources and file formats in databases with automated annotations*). These applications have the potential of enhancing and streamlining internal agencies’ processes. They are developed collaborating with expert AI research groups.

In summary, transparency is about empowering regulatory agencies to acquire all the information they need to make informed decisions. This is the first condition for regulators to be able to assess AI use in drug development. They nevertheless have, of course also to take further actions to keep on ensuring the safety of patients treated with drugs in which AI has been used during one or several steps of their development. Since data, models and applications utilized when applying AI tools depend on the drug lifecycle step (Figure 1A), we propose here stepwise regulatory actions or adaptations. We also show how AI may help optimize and expedite internal regulatory agencies’ processes, simplify review timelines, and improve efficiency while maintaining the highest safety standards (Figure 1B).

### Challenges and corresponding proposals in adjusting to AI’s use across the drug lifecycle

For the whole drug lifecycle, EMA suggests a risk-based approach so that developers preemptively and proactively establish the risks that need to be monitored and/or mitigated (EMA, 2023a). The FDA and the MHRA address mainly the use of AI in medical devices and for digital health technologies (sensors, wearables, etc.) (federalregister, 2023; MHRA, 2024b; MHRA, 2024a). Different centers within the FDA (CBER, CDER, CDRH, and OCP) also collaborate to leverage AI and other advanced technologies to enhance the regulation of medical products (FDA, 2024b). Overall, up to now, no regulatory recommendation proposal for drug development has been published. Stepwise (drug discovery, non-clinical and toxicity, translational and clinical research, pharmaceutical manufacturing, and pharmacovigilance), specific regulatory challenges are therefore delineated here, together with points to consider and possible future adaptations, which are presented in Table 1.

**TABLE 1:** Regulator’s considerations and possible regulatory actions by subject of potential interest in the different steps of the drug lifecycle.

Drug lifecycle steps	Data related subjects	Models and applications related subjects	Considerations/possible regulatory actions
A- Drug discovery	Use of chemical and pharmacological big data Genetic association analysis, pathway mapping, molecular docking, and signature profile matching data	Mode of action/AI prediction Ranking of promising drug compounds Drug repurposing	If determinant in the body of evidence: Assessing relevance of data used/ability to check comprehensiveness and relevance models used
B- Non-clinical testing and toxicity prediction	Use of toxicological big data	Specific models for toxicity prediction (ex: RASAR)	Assessing data used: ability to check comprehensiveness and relevance New regulatory standards for toxicity prediction if used in animal full replacement methods Evolution of pharmacopeial monographs
C-Translational and clinical research	Synthetic data	Digital twins	Ability to check relevance of the synthetic data
	EHR, data from various sources (omics, imaging, pathology, etc.) real-world data	Clinical trial design optimization	Checking/assessing data used Relevance of potential regulatory action to be determined
	EHR, use of real-world data	Selection of patients	Expected regulatory adaptations needed
	Use of clinical big-data (imaging, pathology, omics, etc.)	Analysis of clinical trials	Enhancement of classical routine clinical trial analysis Relevance of potential regulatory action to be determined. Use of clinical big-data already in research hospitals (clinical trials)
D- Pharmaceutical manufacturing, QC, QA	Use of in-house firms’ data	Specific models and applications: process design, optimization, in-control advanced process control, used before process or on-site	Potential confidentiality issues. Regulations to be adapted Knowledge of model precision, accuracy, and state-of-the-art. For continuous production or models used in live production control, specific training of inspectors Evolution of pharmacopeial monographs
E- Pharmacovigilance (PV)	Usual and “Non-conventional” big-data use: social networks, medical forums	Early or wider detection of PV signals/detection from more sources and big-data	Validate relevance and usefulness of data Check and test relevance of detections: part of model training. Might be put in routine production soon

In the race for innovative therapies, AI emerges as a powerful ally in drug discovery. Only one wholly AI-designed drug has entered clinical trials thus far (Generative Artificial Intelligence for Drug Discovery, 2024), and regulatory agencies are not mandated to assess the methodologies used unless they contribute to the overall body of evidence. However, as AI models become integral to drug design, a dialogue between regulators and developers becomes imperative to ensure transparency and understanding of model performances as regards their predictions’ accuracy and reproducibility (Table 1 A). Additionally, AI holds promise for accelerating drug repurposing efforts, leveraging big data analysis to identify new medical indications for existing drugs with unprecedented speed and precision (Zong et al., 2022).

In regulatory science, and specifically in non-clinical testing and toxicity prediction, AI tools have great potential to predict safety outcomes, but their suitability remains to be determined. First of all, these tools offer a promising avenue to potentially reduce or even replace the traditional reliance on animal testing, which is a powerful incentive (EMA, 2023a). Notably, the FDA Modernization Act 2.0 in the United States takes a stride forward by curbing the mandatory use of animal models for toxicity predictions (Han, 2023). AI non-clinical models draw from a rich diversity of data sources—from *in vitro* and *in vivo* experiments to expansive databases—employing diverse algorithms and machine learning techniques (Maertens et al., 2022). Toxicity predictions generated using AI (machine learning on relevant biological, chemical, or toxicological data) are inherently probabilistic and contingent upon the quality and quantity of the input data, but they have great potential (Mayr et al., 2016; Wu et al., 2021; Maertens et al., 2022). However, rigorous assessment of the data and models used and potential adjustments to regulatory frameworks will be necessary in the long run (Table 1 B) (Paul et al., 2021). Several efforts have been made or are underway to curate and reliably annotate toxicological databases (Lea et al., 2017; Nair et al., 2020; Wu et al., 2023).

In translational and clinical research, several regulatory projects regarding AI use are currently led by the FDA and EMA (EMA, 2023a; FDA, 2024c), underscoring the burgeoning potential of AI in these domains. During the COVID-19 pandemic, AI played a crucial role in accelerating vaccine trials. Companies like Moderna and Pfizer used AI to design trials, monitor patient data, and streamline regulatory submissions. AI tools helped identify suitable trial participants more quickly, designed adaptive trial protocols that adjusted in real-time based on interim results, and monitored adverse events to ensure participant safety. This use of AI contributed to the unprecedented speed at which COVID-19 vaccines were developed and approved (reviewed in (Sharma et al., 2022)). However, the current absence of regulations in this domain raises pertinent questions, highlighting the pressing need for new oversight (Arora and Arora, 2022). Take, for instance, the digitization of clinical trials—an innovative approach leveraging data from electronic health records (EHR), routine medical exams, and various diagnostic tests. This digital transformation not only streamlines patient selection but also opens doors for broader trial participation. Yet, navigating the complexities of data management in these trials necessitates transparency in AI algorithms (Kasahara et al., 2024), and several open questions remain (Table 1 C).

In drug manufacturing, AI tools are also revolutionizing various aspects, from process design and scaling up to advanced control and fault detection. Both the FDA and EMA are actively crafting recommendations in this domain (EMA, 2023a; FDA, 2024a). While the full extent of AI’s impact is yet to be realized (consultations are ongoing), it’s evident that the field is rapidly expanding (Table 1 D). Given that these techniques primarily originate in industrial sectors, fostering closer collaboration between manufacturers and regulators is imperative. Notably, the real-time application of these methods on the factory floor poses unique challenges, necessitating robust regulatory frameworks and onsite inspections for compliance.

In pharmacovigilance, AI is gaining traction as a potent tool for enhancing drug safety monitoring. The EMA’s reflection paper acknowledges its significance, while the FDA’s discussion paper delineates its role across case processing, evaluation, and automated submissions prior to individual safety report submissions (EMA, 2023a; FDA, 2024c). In pharmacovigilance, regulators already take advantage of AI techniques to better deal with big data from various sources. Pharmacovigilance is therefore the field in which the use of AI is now most mastered and is currently used by regulatory agencies (Martin et al., 2022; Routray et al., 2020; Actualité, 2024), which may and should establish collaborations with academic research laboratories to use AI for specific projects with low-level or early detection signals.

More specifically, AI is used here for improving data analysis from institutional databases (World Health Organization’s Vigibase, EMA’s Eudravigilance, FDA’s Adverse Event Reporting System (FAERS), etc.) by developing AI algorithms better than the classic statistical ones. AI may also be used for improving data quality in databases (symbolic AI), allowing better groupings before analysis, increasing the number of cases by developing AI tools to collect more data from physicians or patients; or using other sources (EHR or social media) (Table 1 E).

Another key aspect is that as the landscape of drug development evolves, it’s becoming increasingly clear that regulatory agencies will need to bolster their expertise in AI. This demand for AI-specific skills varies depending on the stage of drug development, so specific stepwise upskilling of assessors will be required. Indeed, the evaluation of the specific data, applications, and models used will be needed for preparing corresponding scientific assessment reports (see specific data sources and applications in Table 1).

### Seizing AI opportunities: optimizing regulatory processes

Regulatory agencies have to deal with amounts and sources of data that are increasingly diverse and massive (raw data reports, real-world data, images, tables, EHR, etc.). Beside the drug lifecycle, AI applications would also increasingly find their place in regulatory assessments (Figure 1B). Recent published advances come from the EMA (Jornet, 2024). First, natural language processing and optical character recognition tools may be used to annotate, extract, and categorize relevant data from various sources submitted to these agencies (files for clinical trials and marketing authorizations, including text, tables, and images). The output will be implemented in AI-amenable databases. An application could then assess the contents and notify the relevant assessors. This would save time, improve reproducibility, and reduce errors (sparing humans low-added value and repetitive tasks). EMA uses AI to support the validation of variations by flagging missing documents, detecting dissimilarities, and automatically identifying changes. AI tools can also find personal data, compare documents, do triage, and perform automated literature reviews. At the European level, there are also several other projects aimed at challenging and furthering AI use in a regulatory setting. (EMA, 2023b; bundesgesundheitsministerium, 2024; Regulatorische, 2024). A new NLP approach for harmonization of European medicinal product information has also recently been published (Bergman et al., 2022).

In the regulatory setting, AI tools may be used to categorize and annotate texts from various sources and help implement progressively a collective memory to compare files, perform pre-analyses, and produce knowledge graphs (making comparisons easier). They could also, theoretically, help as regulatory assessment assistants in the near future.

## Perspectives and conclusion

Today, it is quite difficult to gather accurate data on AI use in the lifecycle of drugs in published data except at the clinical trial stage. This shows that information about AI use for health topics or in health products is not readily and easily accessible. The first major factor for relevant assessment by regulators of these tools and applications is transparency, which goes with explainability using relevant tools [see above and (Lundberg and Lee, 2017)]. The second will be adapting current recommendations for developing new regulatory guidelines for AI use in the healthcare setting and collaborating with researchers, physicians, and the industry to improve the relevance of these guidelines. This could foster transparency, which regulators, the public, and health professionals demand (Vellido, 2019). Other factors that have to be considered are the inherent complexity of AI models and their “black-box” nature (Rudin, 2019) and concerns about data privacy and security (Price, 2017). These are the main factors that may question stakeholders, such as patients and the general public. The international conference on harmonization (ICH) has recently addressed the use of AI and modeling for some topics related to the quality of drugs (e.g., product dissolution and *in vivo*/*in vitro* relationships/correlations, purge and fate of impurities, container/closure integrity, etc.) (ICH, 2021), which would impact the ICH M7 guideline. The ICH M15 concept paper, “Model-Informed Drug Development General Principles Guideline,” also considers future approaches such as machine learning and AI (M15, 2022). The ICH is therefore already considering the use of AI in drug development.

Applications of AI for internal processes at agencies also have great potential. To this end, there is important work to be done at regulatory agencies for selecting and validating the data incremented in the databases that will be useable to create the collective memory to be used for better and quicker assessments. In any case, this will require both collaboration with AI academic research laboratories, and acquiring internal competencies.

As we look into the future of drug regulation amidst the burgeoning era of AI, several critical questions subsist.

First of all, it remains to be determined what concrete steps can be taken to ensure that all stakeholders are involved (including patient associations in the larger frame of health democracy) in the utilization of AI tools in drug development. This health democracy setting will be important to better take into account potential ethical issues, such as potential patient selection and monitoring of clinical study biases. Another important point for the future will be to determine how regulatory bodies can navigate the complexities of AI models, while ensuring all corresponding ethical aspects. The ethical implications of using AI in drug development, include potential bias in AI models, informed consent, and patient autonomy. The use of AI may also raise privacy concerns like data privacy issues, data security, patient confidentiality, and compliance with regulations like GDPR. Potential solutions to these concerns are to design strategies like implementing robust data anonymization techniques, ensuring diverse and representative data sets to reduce bias, involving all stakeholders (including patient representatives), establishing transparent AI model validation processes (transparency, explainability), and adhering to local and international ethical guidelines and frameworks. Looking ahead, international collaboration among regulatory authorities will be instrumental in developing common responses and standards for evaluating AI technologies in pharmaceutical development. By harnessing the collective expertise and resources of global stakeholders, regulators should forge an adaptive framework that fosters transparency, innovation, and patient-centric outcomes.

## Data Availability

The original contributions presented in the study are included in the article/Supplementary Material, further inquiries can be directed to the corresponding author.

## Members of The Scientific Advisory Board of ANSM

Joël Ankri (Chairman), Janine Barbot, Robert Barouki, Éric Bellissant, Patrick Castel, Patrick Chaskiel, Nicolas Clere, Christiane Druml, Sofia de Oliveira Martins, François Eisinger, Éric Ezan, Catherine Gourlay-France, Jamila Hamdani, Walter Janssens, Marie-Christine Jaulent, Maria Emilia Monteiro, Sylvie Odent, Fred Paccaud, Dominique Pougheon, Vololona Rabeharisoa, Victoria Rollason, Valérie Sautou, Jean-Pierre Thierry, Jean-Paul Vernant.
